# Unnecessary hysterectomies and our role as interventional radiology community

**DOI:** 10.1186/s42155-020-00138-x

**Published:** 2020-07-14

**Authors:** Gregory C. Makris, Saqib Butt, Tarun Sabharwal

**Affiliations:** 1 Vascular and Interventional Radiology Department, Guy’s and St Thomas’ Hospital, NHS Foundation Trust, Westminster Bridge Road, London, SE1 7EH UK; 2 Alfa Institute of Biomedical Sciences, Neapoleos 9, Marousi, Athens, Greece

**Keywords:** Hysterectomy, Uterine artery embolization

To the Editor,

We read with interest the article by de Bruijn and colleagues(de Bruijn et al. 2019) who reported that despite the inclusion of uterine artery embolization (UAE) in the management of heavy menstrual bleeding in Dutch national guidelines, the UAE to hysterectomy ratio remained low at 6.9%, with no increase following the introduction of these new guidelines.

Such low UAE rates are not isolated to the Netherlands. Spanish data suggests approximately 145 UAEs performed annually in a population of over 44 million,(https://interventionalnews.com/uae-surgery-disparity-worldwide/?hilite=%27fibroid%27%2C%27embolization%27 n.d.) whilst in France an average of 2000 UAEs are performed per annum compared to 40,000 hysterectomies.(https://interventionalnews.com/uae-surgery-disparity-worldwide/?hilite=%27fibroid%27%2C%27embolization%27 n.d.) Data from the national heavy menstrual bleeding audit in the UK revealed that of the 6195 women who were treated surgically, only 129 (2%) had UAE.(Geary et al. 2019) A similar picture persists outside of Europe. Australian Medicare data reveals an average of less than 200 UAEs performed per year,(Al-Fozan et al. 2002) whilst in the USA, 65 times more hysterectomies are performed than UAEs.(Narayanyan et al. 2017) This is concerning, especially in light of trial evidence showing that two thirds of women treated with UAE remain hysterectomy free at 10 years.(de Bruijn et al. 2016) In addition, there is no difference in patient satisfaction between UAE and hysterectomy at 5 years, whilst the duration of procedure, length of hospital stay and time taken to resume normal activities may be shorter in UAE relative to hysterectomy or myomectomy.(Gupta et al. 2014) Further supporting treatment with UAE, twenty year follow up cohort studies demonstrate an increased risk of metabolic and cardiovascular conditions following hysterectomy, especially in women under 35.(Laughlin-Tommaso et al. 2018)

Patient awareness of UAE remains concerningly low. A report by the Society of Interventional Radiology in 2017 found that 62% of women had never heard of the treatment.(The fibroid fix 2017) Of the women who were aware of UAE, almost three quarters did not first hear of it from their gynaecologist, suggesting that despite the wealth of high quality evidence, gynaecologists are failing to offer this safe, effective, minimally invasive treatment to their patients.

We believe that at a local level this could be remedied by involvement of interventional radiologists at gynaecology multidisciplinary team (MDT) meetings and the patient counselling process. The regional situation in the UK is complicated by funding for procedures being devolved to clinical commissioning groups, allowing a postcode lottery to exist whereby UAE is funded in some regions but not others. With increased awareness amongst patient groups and general practitioners in addition to the large body of evidence demonstrating clinical and cost effectiveness, the case for funding UAE is a strong one. Nationally, closer collaboration between interventional radiology and gynaecology societies would also help to increase awareness amongst referring gynaecologists.

In short, the interventional radiology community must continue to address the injustice of chronic underutilisation of this minimally invasive, safe, clinically and cost effective procedure. Engaging with relevant stakeholders and lobbying at social and political levels is crucial for the success of this endeavour.

## Data Availability

Not applicable.
